# Aging 2.0: Health Information about Dementia on Twitter

**DOI:** 10.1371/journal.pone.0069861

**Published:** 2013-07-26

**Authors:** Julie M. Robillard, Thomas W. Johnson, Craig Hennessey, B. Lynn Beattie, Judy Illes

**Affiliations:** 1 National Core for Neuroethics, University of British Columbia, Vancouver, British Columbia, Canada; 2 Department of Electrical and Computer Engineering, University of British Columbia, Vancouver, British Columbia, Canada; Edinburgh University, United Kingdom

## Abstract

Online social media is widespread, easily accessible and attracts a global audience with a widening demographic. As a large proportion of adults now seek health information online and through social media applications, communication about health has become increasingly interactive and dynamic. Online health information has the potential to significantly impact public health, especially as the population gets older and the prevalence of dementia increases. However, little is known about how information pertaining to age-associated diseases is disseminated on popular social media platforms. To fill this knowledge gap, we examined empirically: (i) who is using social media to share information about dementia, (ii) what sources of information about dementia are promoted, and (iii) which dementia themes dominate the discussion. We data-mined the microblogging platform Twitter for content containing dementia-related keywords for a period of 24 hours and retrieved over 9,200 tweets. A coding guide was developed and content analysis conducted on a random sample (10%), and on a subsample from top users’ tweets to assess impact. We found that a majority of tweets contained a link to a third party site rather than personal information, and these links redirected mainly to news sites and health information sites. As well, a large number of tweets discussed recent research findings related to the prediction and risk management of Alzheimer’s disease. The results highlight the need for the dementia research community to harness the reach of this medium and its potential as a tool for multidirectional engagement.

## Introduction

The emergence of “Web 2.0″, a platform that allows all its users to create and modify online content on an ongoing basis, has led to new ways of accessing and exchanging health information. Moving away from passive consumption of top-down dissemination efforts, online users are now actively engaged in sharing health-related knowledge and experience. With a growing population of older adults and the associated increase in the prevalence of age-related dementias such as Alzheimer disease, the dissemination and the quality of information relating to aging and dementia have the potential to significantly impact the health of older adults and the economic burden associated with their care. In this new age of information technology, it becomes imperative to empirically assess the information exchanged about aging and dementia on social networks to inform the optimization of the delivery and the accessibility of this information.

The demographics of the developed world are rapidly shifting towards an increase in the number of older adults. As a result, the prevalence of Alzheimer disease and other age-related diseases of the central nervous that are characterized by dementias are projected to rise significantly - by 2050, it is estimated that 1 in 45 Americans will be afflicted by Alzheimer disease alone [1]. The social, health, and economic burden will be substantial [2]. While there are currently no preventive or disease-modifying therapies for Alzheimer disease [3], lifestyle interventions can play a role in prevention [4]. These interventions, such as changes to diet and exercise regimens, have the potential to significantly impact overall public health [5]: a delay in the onset of Alzheimer of just two years today would lead to a decrease of 2 million cases 50 years from now [1].

As both non-pharmacological and eventual pharmacological interventions to promote brain health may have such a major role to play in public health, and as new findings about Alzheimer disease emerge from research, communication becomes a priority. Alongside traditional media, the Internet is rapidly growing as an important source of health information that will undoubtedly impact decision-making [6]. Older adults are open to using these technologies for health information [7]: one-third of older adults are already using the internet to search online for information about health [8], and this number is growing steadily [9].

Social media, in particular, offers new possibilities for communication, and is a growing tool for engagement about health and disease. Web- and mobile-based applications of social media are powerful tools for the dissemination of health information as they can reach a broad audience in a very short period of time, are easy and affordable to access and use, and cater to a large variety of audiences. While social media is only about 15 years old and very large-scale applications such as Facebook are not yet 10 years old, already these applications impact how doctors and patients interact [10] and are changing health communication patterns [11]. By harnessing the power of global, widely used user-generated content, social media allows for new channels of information that range from traditional top-down dissemination to peer-based discussion and exchange, each with their own distinct thematic content.

In this era of social media, Twitter has emerged as one of the most popular microblogging platforms that allows users to send and read short text-based messages limited to 140 characters. There is an increasing interest in analyzing social media data, and Twitter in particular, to gain insights about online activities and their impact [12], [13]. While research into Twitter is still in its infancy, creative new uses of this platform are clearly emerging. Twitter content has been used in a wide range of contexts, from the detection of real-time events such as earthquakes [14] to the evaluation of word-of-mouth communication about corporate brands [15]. The potential impact of Twitter on offline behaviors has been demonstrated in the context of emergency events such as hurricanes and floods, where Twitter is used for information broadcasting and brokerage [16], [17]. While little data focuses specifically on microblogging and health, Twitter content has been studied for tracking flu empidemics [18] and to assess public misunderstandings surrounding antibiotic use [19]. A number of studies suggest that analyzing content such as tweets can be a productive way to evaluate discourse surrounding health and disease [18], [19], though no work currently exists on the discussion about aging and dementia on Twitter.

The goal of the present study is to fill this knowledge gap through the characterization of the thematic content of dementia-related tweets and the evaluation of the nature of health information exchanged through this novel and powerful communication medium.

## Methods

### Design

We conducted a cross-sectional survey study through content analysis of microposts on the online social media platform Twitter for a period of 24 h starting February 15 2012 at 3∶35 pm. The methodology was developed for this study and draws upon previous methods for analyzing social media content [18], [19].

### Study Setting and Search Strategy

We applied content analysis to publicly available microposts (“tweets”) posted to the online site Twitter. Twitter was chosen over other microblogging platforms based on worldwide popularity – it currently ranks as the most visited microblogging platform with the most active users (500 million). We created an automated program utilizing the Twitter API to search for English-language tweets containing the words “dementia” or “Alzheimer”. Data fields for users, user information, date and time, and tweet content were parsed and stored. Exact duplicates were automatically removed from the database. Twitter users were not contacted for this study and no attempts were made to access information that users set as private.

### Coding and Intercoder Reproducibility

Search results were randomized and 10% of the tweets were retrieved for analysis.

Using an initial set of 100 random tweets containing the search terms, a pilot analysis was conducted and broad categories were established to form a coding guide using an emergent coding strategy. The coding guide was then refined into its final iteration through discussion between researchers. The coding structure was developed to capture the salient thematic features of the sample with a focus on the salient information as established during the preliminary phase of the coding guide development. Individual codes were applied to each complete tweet as the unit of analysis. We used a rich coding strategy, allowing multiple categorizations of individual tweets [20]. The final coding guide comprised the following major categories, as applicable: 1) type of user (e.g., physician, corporation, patient); 2) content type of link (e.g., news, commercial, advocacy); 3) content type of the tweet (e.g., research findings, news story, humor, personal event); and 4) aspects of dementia (e.g., prevention, prediction, diagnosis, treatment). Research studies uncovered under category 3 were further coded according to their date and publication status.

To measure impact, we also carried out a second analysis of the top users in our sample as defined by how many “followers” are reached by tweets from each “top user”. Top users were characterized as those with the most followers at the time of the study.

### Statistical Analysis

We used descriptive statistics to characterize the composition of the sample.

## Results

### Sample

9,200 unique tweets containing one of the keywords were generated in the target 24 hours period. We randomized this sample and extracted 10% (n = 920) for analyses. From our sample of 920 tweets, we also created a subsample containing 100 tweets generated by the top users. The top users were characterized by having the most followers at the time of the study. The number of followers for top users at the time of the data collection ranged from 3,422 to 133,759.

### Users Tweeting about Dementia

**Figure 1:**
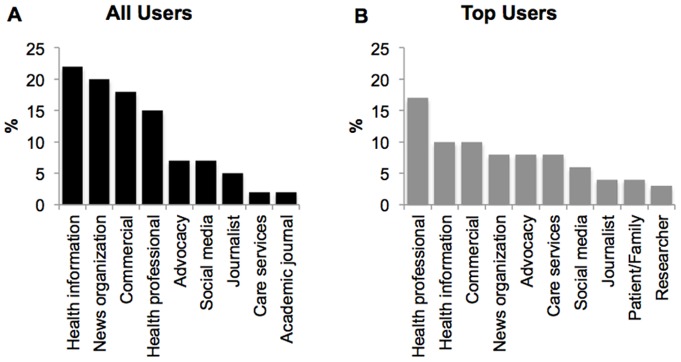
Types of users sharing information about dementia on Twitter. A. All users sample. B. Top users sample.

Partially identifying information, based on publicly available, user-provided data on profile pages, was available for 844 users of the full 920 sample. Of these, we were able to access sufficient information from 460 users to characterize their user type: categories of users included health professionals (17%), commercial entities (10%) and health information sites (10%) (Figure 1). In contrast, four types of users dominated the top user sample: health information sites (22%), news organizations (20%), commercial entities (18%) and health professionals (15%). This difference between the types of users in our total sample and in our top users sample highlights the fact that users tweeting about dementia that reach a broader audience tend to be organizations, not individuals.

### Web Links Tweeted about Dementia

In the full sample, 77% of tweets contained links, compared with 92% for the subsample of top users.

**Figure 2:**
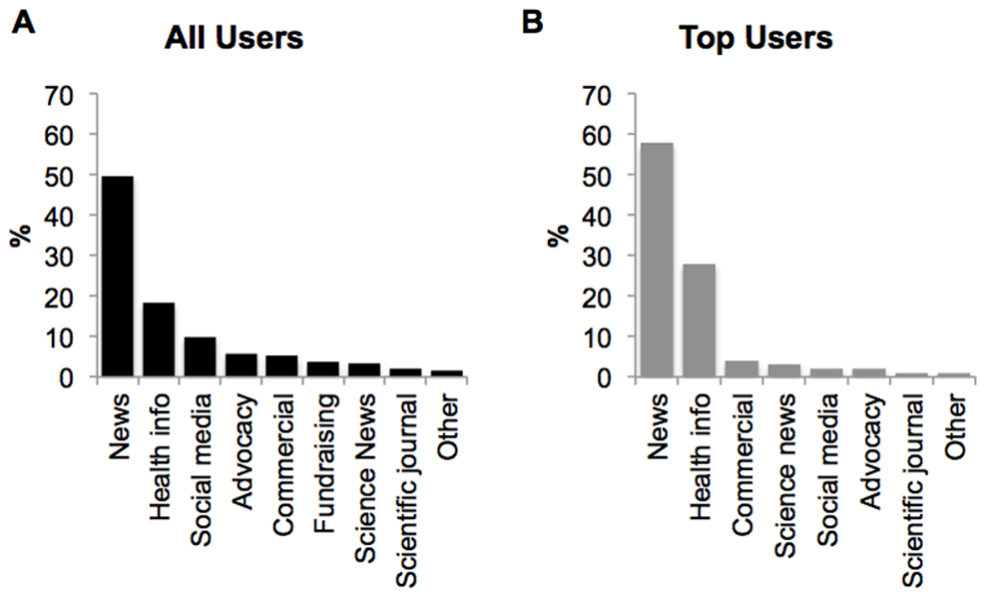
Website links in tweets about dementia. A. All users sample. B. Top users sample.

We followed each of the links and found two main types of linked websites: major news (all users: 50%, top users: 58%) and health information (all users: 18%, top users: 28%). Other linked websites referred to various other types of sites such as social media, advocacy, commercial, fundraising, science news, scientific journals, government, and personal sites (Figure 2).

### Content Tweeted about Dementia

**Figure 3:**
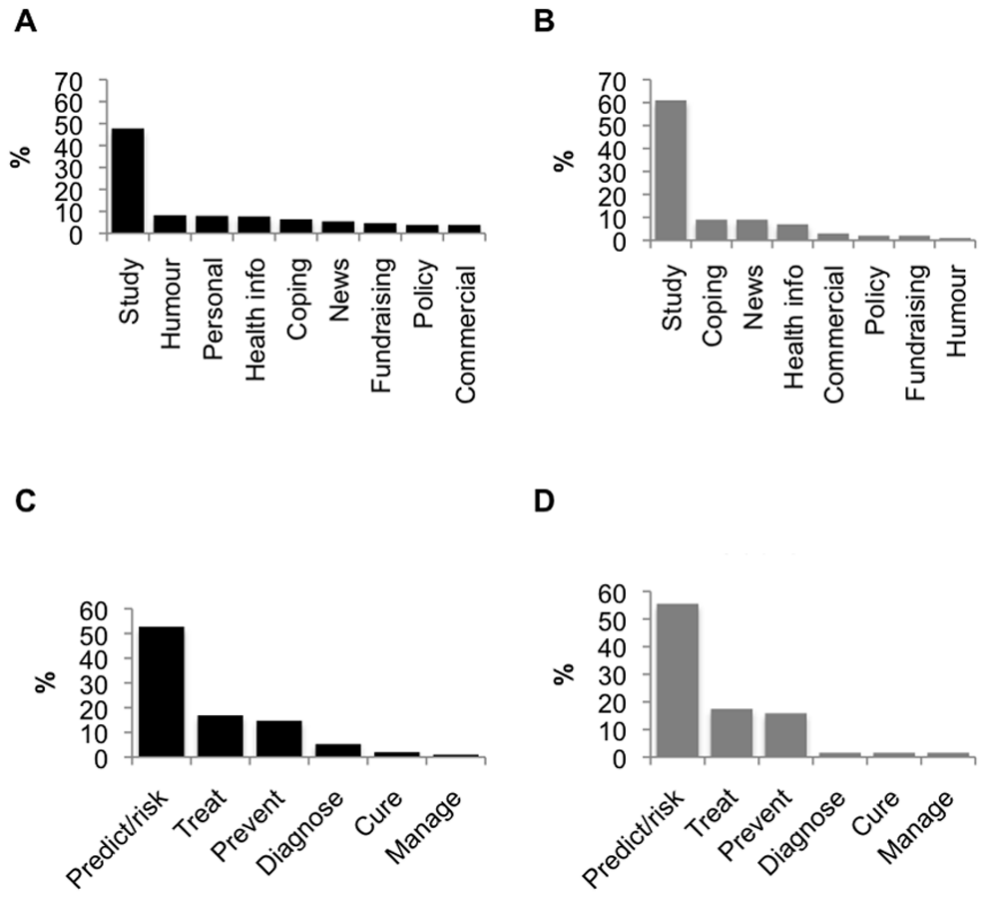
Content shared on Twitter. A. General content in sample of all users. B. General content in sample of top users. C. Dementia-related content in sample of all users. D. Dementia-related content in sample of top users.

Tweets about research occurred most frequently (all users: 48% (n = 441/920), top users: 61%; (Figure 3, A and B).) and referred to 38 distinct identifiable research studies. Of these research studies mentioned, 76% consisted of peer-reviewed original research. The studies were current: a majority (58%) were published in the two weeks preceding this study’s data collection and the oldest study dated back to 2008. The majority of the studies originated from the United States (61%).

Of the tweets about primary research, one study about the link between walking speed and dementia risk (see Table 1) accounted for 32% of the mentions. The other two most discussed studies were about sleep and risk of Alzheimer disease (17%) and about the impact of curcumin, a member of the ginger family, on a fruit fly model of Alzheimer disease (10%). Of tweets specifically about an aspect of Alzheimer disease or dementia, a majority (all users: 53%, top users: 56%) were about prediction or risk management (Figure 3, C and D).

**Table 1:** Top research studies discussed in tweets about dementia.

Code	Finding	Publication status	Percentage
Walking speed	Slower walking speeds were linked to a higher riskof dementia	Academy of Neurology annual meeting	32%
Sleep	Poor sleep leads to amyloid plaque build-up	Academy of Neurology annual meeting	17%
Curcumin	Curcumin prolongs life and enhances activity of afruit fly model of AD	PLOS ONE, March 2012	10%
Cancer	Cancer drug reverses cognitive deficits in AD mice	Science, March 2012	9%

## Discussion

Content analysis of tweets from a major online social media platform provides insights into how online users share information about dementia and the type of information shared. The results show that: 1) the push of information about dementia on Twitter comes mainly from users who identify as health professionals, health information sites, news organizations and commercial entities; 2) most tweets contain links to news and health information sites; and 3) recent studies about prediction and risk management were at the forefront of the discussion. We uncovered differences between our overall sample and our top users. The distribution of types of users clearly suggests that top users consist mostly of large entities such as news organizations, and not individuals. We also found that top users send out more tweets containing links. This difference may be due to the fact that our sample from all users contains more individual users, which collectively may generate more tweets that are personal in nature, compared with organizations. In our sample, no organization posted content characterized as personal. It is also worth noting that Twitter enforces a 140-character limit on posts, which limits the extent and style of information in tweets.

Recent survey data shows that older adults are increasingly online: 58% of American older adults aged 65 years or older are using the Internet, and this figure increases to 85% for older adults aged 50 to 64 (Pew Research Center’s Internet and American Life Project Surveys, 2010). The Internet offers broad access to health information [21] in a way that is both interactive and anonymous [22]., making the Internet an attractive medium to seek such information. Of adult Internet users, 80% capitalize on the opportunity to look for health or medical information online. While little data exist about information-seeking behaviors specific to aging and dementia, the changing characteristics of the population and the rising prevalence of Alzheimer disease and other dementias are resulting in an increase in Internet resources for these conditions [23]–[25].

Despite the value of the new knowledge generated by this work, we appreciate its limitations. Social media, like other tools to assess public opinions, lends itself to selection bias, and it is difficult to assess whether the study population represents the sampling population [26]. The inherent anonymity of users makes it impossible to verify the authenticity of content or to undertake statistical analyses by age, gender or other demographic characteristic to establish correlates of information-sharing behaviors. A time period of 24 h is a snapshot of activity only, and the timing of this period (in this study, during a weekday) could have implications for the demographics of the user sample. For this study “retweets”, which consist of reposts of other users’ tweets, were excluded to ensure the data was an accurate representation of newly generated content on Twitter. Future work looking at retweets might provide additional information about how information is disseminated on Twitter. Our sample of top users is defined by their number of followers, which can be a misleading metric as organizations may have a wider existing network and greater resources to expand that network. Finally, while Twitter boasts high traffic and a relatively broad demographic, it is not the only social media platform used to share information. Discussion forums, for example, leave more room for conversation about personal experiences.

Nevertheless, Twitter is one of the top ten most visited websites on the Internet and has over 500 million active users around the world (Alexa, retrieved December 3 2012). It has been shown to significantly impact communities [17], [27] and represents an important platform for the dissemination of information about health. Our findings here support and extend the results of these prior reports. We show that the discussion about dementia on Twitter is dominated by links to third-party sites containing information, not by personal anecdotes or experiences. Therefore, while Twitter users engage in information-sharing through social media, their sources of information remain based in traditional media. As coverage of dementia in traditional media is not without its pitfalls [28], the findings highlight the potential for social media to perpetuate and perhaps even promote sources of information of unknown quality. Moreover, caregivers and relatives of dementia patients may be more likely to be the recipients of the information posted on Twitter, and their use of this information may differ from that of people with dementia. Further work will be needed to assess whether these phenomena have a significant impact on Internet users’ trust and use of different sources of information.

The results also show that during the time of the study a dominant theme in the discussion of dementia on Twitter was risk. Several stories about the correlation between certain behaviors (e.g., walking speed, amount of sleep) and the risk of developing Alzheimer disease are interpreted to reveal insights about the prediction of the disease. The potential for misinterpretation of correlation versus causation can occur at all levels in the chain of communication, from the peer-reviewed article to the news report or the individual Twitter user. While the evaluation of the quality of the sources of information about dementia was outside the scope of this research, future work in this area could provide additional insights on the sources of misinformation. Regardless of the origin of the information about dementia and, more broadly, about health, the possibility of perpetuating misunderstandings on Twitter highlights one of the risks of multidirectional communication on social media platforms [19]. Further work will be needed to address how these risks weigh against potential benefits such as the promotion of positive behavior changes.

Across health and illness, social media is reshaping health care, acting as a powerful new way for stakeholders to interact and share information [10]. The results here confirm the engagement of a wide range of stakeholders, ranging from physicians to patients, on Twitter. As a whole, social media applications hold potential to enhance the health of its users. Interacting through social media can increase social support and feelings of connectedness [29], [30] as well as lead to a sense of empowerment in patients [31]. Interactive Internet applications also increase the democratic aspect of information-sharing, which in turn leads to a more patient-centered experience [10]. Direct efforts to promote health through social media have a broader reach than traditional media and for some conditions, such as smoking cessation and dietary interventions, that are also intertwined with aspects of aging and dementia, have been shown to have a significant impact [32]–[34].

The benefits of social media are not untouched by risk. Far-reaching, easily accessible platforms can lead to the wide dissemination of misinformation [35]. Our results show a strong interest for the prediction of Alzheimer disease based on lifestyle metrics, which may be resulting from the misinterpretation or the oversimplification of findings from scientific studies. As well, there is still evidence of a double-divide (age and socioeconomic status) when it comes to accessing the Internet, though this divide seems to be diminishing over time [36]. Faced with the shortcomings of health information online [37], [38], the scholarly community must pay close attention to what types of information is available online and how to mitigate the potentially negative impact of this information on public health. One solution to this problem calls for the involvement of researchers in the dissemination of their findings [39], as well as their engagement in online social media.

Twitter represents a spontaneous form of public engagement, and we report on the dominating sources of information and dementia themes discussed here. The present work and future studies in this field are critical to inform both research and medical communities about the current state of information-sharing behaviors regarding dementia, and highlight the need for careful, evidence-based research about the fast-moving developments in this field.
